# Complete genome sequence of *Roseomonas* sp. WA12 isolated from the rhizosphere of *Artemisia ordosica*

**DOI:** 10.1128/mra.00012-26

**Published:** 2026-03-13

**Authors:** Bo Yuan, Siyi Wang, Yu Hong

**Affiliations:** 1 College of Life Science and Technology, Inner Mongolia Normal University 71203 , Hohhot, Inner Mongolia Autonomous Region, China; 2 Key Laboratory of Biodiversity Conservation and Sustainable Utilization in Mongolian Plateau for College and University of Inner Mongolia Autonomous Region 71203 , Hohhot, China; DOE Joint Genome Institute, Berkeley, California, USA

**Keywords:** complete genome, *Roseomonas* sp. WA12, *Artemisia ordosica*, rhizosphere-inhabiting microbes

## Abstract

We report the complete genome sequence of *Roseomonas* sp. WA12, a strain isolated from the rhizosphere soil of *Artemisia ordosica*, which is grown in Jungar Banner, China. The genome consists of a circular chromosome (4,787,499 bp; GC content: 69.70%) and three circular plasmids, which are named plasmid A (731,044 bp; GC: 66.04%), plasmid B (219,984 bp; GC: 65.08%), and plasmid C (44,809 bp; GC: 67.83%), respectively.

## ANNOUNCEMENT

Currently, numerous agricultural lands worldwide are experiencing varying degrees of water scarcity, with the intensity and persistence of such shortages influenced by climate change (1). Insufficient water supply can rapidly lead to plant dehydration, subsequently affecting crop growth and development (1). However, rhizosphere bacteria may promote crop growth. The genus *Roseomonas*, characterized by its metabolic diversity and widespread distribution (2, 3), plays essential roles in mediating key ecological and biogeographical processes (4). *Artemisia ordosica*, a dominant constructive species in the Mu Us Sandy Land of northwestern China, makes up to 31% of the land’s vegetation cover and plays a crucial part in preserving the structural stability of the regional ecosystem (5). This study aims to obtain the complete genome of *Roseomonas* sp. WA12 to further explore the beneficial interactions between this strain and its plant host.

*Roseomonas* sp. WA12 was isolated from the rhizosphere soil of *Artemisia ordosica*, which grows in Jungar Banner (40.17°N, 111.07°E), Inner Mongolia Autonomous Region, China. A 1 g soil sample from *Artemisia ordosica* roots was homogenized in 9 mL of normal saline. After centrifugation to sediment soil particulates, the supernatant was spread-plated on R2A medium (6) and incubated at 28°C for 48 h. Single colonies were isolated through three successive rounds of single-colony streaking on R2A. Multiple isolates were identified as *Roseomonas* via PCR amplification with the specific primer pair 27F/1492R. Among these isolates, strain WA12 was selected for sequencing. A single colony of WA12 was cultured in R2A liquid medium for 36 h, and bacterial cells were harvested from 50 mL of culture by centrifugation. Genomic DNA was extracted using a magnetic bead-based bacterial DNA Extraction Kit (Majorbio, Shanghai, China). Sequencing was performed on both Illumina NovaSeq Xplus platforms and PacBio Sequel IIE (producing HiFi reads). The DNA was sheared into fragments of approximately 400 bp by Covaris M220 Focused Ultrasonicator (Covaris, USA), with the size distribution verified by agarose gel electrophoresis. A sequencing library was constructed using the NEXTFLEX Rapid DNA-Seq Kit (Illumina, USA) and sequenced on the NovaSeq Xplus (Illumina, USA) platform with 2 × 150 bp paired-end reads. Raw data quality control was performed using fastp v0.23.0. For Illumina sequencing, paired-end reads (2 × 150 bp), totaling 9,094,856 reads covering a total of 8,921,026 clean data (Q20, 98.74%; Q30, 93.78%) with 237.46-fold genome coverage, were obtained. For PacBio sequencing, genomic DNA was fragmented at 8–10 kb by the G-tubes method and then sequenced using the SMRTbell prep kit 3.0 (PacBio, USA) for the third-generation library construction. PacBio sequencing generated a total of 53,027 reads with an N50 length of 11,565 bp and an average read length of 11,539.51 bp providing approximately 105.80-fold genome coverage. The PacBio reads were assembled using unicycler v0.4.8 (7) at a sequencing depth of 135×. The resulting genome was polished with Pilon v1.22 (8) to obtain a high-accuracy final sequence. A phylogenetic tree was constructed using MEGA-X 10.0.2 (9). All software was run with default parameters unless otherwise specified.

**Fig 1:**
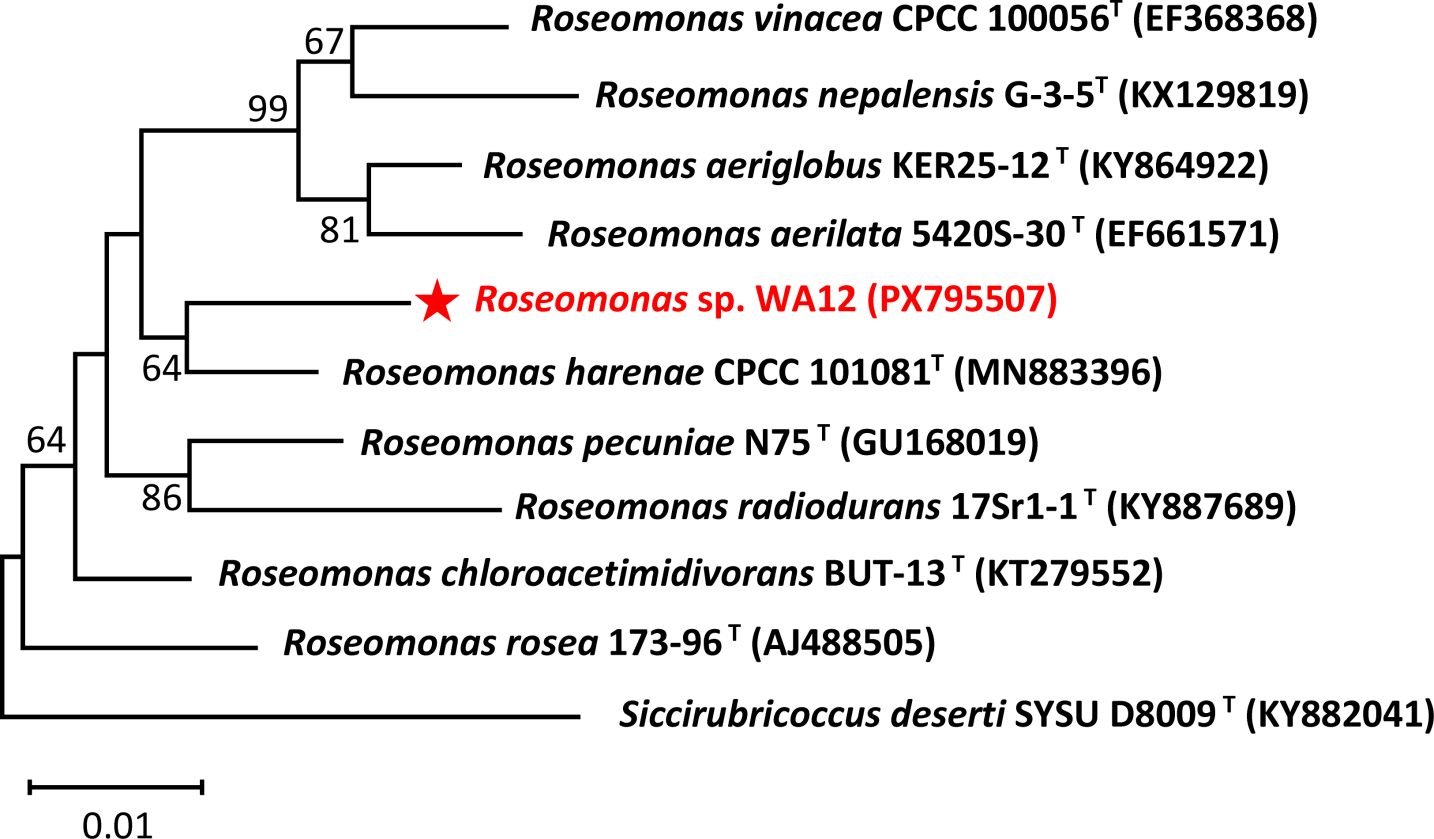
Neighbor-joining tree based on 16S rRNA gene sequences using the Kimura 2-parameter method in MEGA-X 10.0.2, showing the phylogenetic position of strain WA12 within related genera. Bootstrap values were based on 1,000 replicates (values ≥ 50% were shown). *Siccirubricoccus deserti* SYSU D8009^T^ was used as the out-group. Bar, 0.01 changes per nucleotide position.

The genome size of *Roseomonas* sp. WA12 is 5,783,336 bp, containing one circular chromosome (4,787,499 bp; GC content: 69.70%) and three circular plasmids, which are plasmid A (731,044 bp; GC: 66.04%), plasmid B (219,984 bp; GC: 65.08%), and plasmid C (44,809 bp; GC: 67.83%), respectively. The genomic annotation statistics for strain *Roseomonas* sp. WA12 is summarized in Table 1. The length of the 16S rRNA gene is 1,304 bp, and the 16S rRNA phylogenetic tree indicates that strain WA12 belongs to the genus *Roseomonas* (Fig. 1). It shows the highest similarity of 98% with the type strain *R. harenae* CPCC 101081^T^.

**TABLE 1:** Genome annotation statistics of *Roseomonas* sp. WA12

Characteristic	*Roseomonas* sp. WA12
Total bases (bp)	4,787,499
GC (%)	69.70
CDS	5,327
Complete rRNAs (16S, 23S, 5S)	3, 3, 3
tRNAs	50
sRNA	17
CRISPR	7

## Data Availability

The Whole Genome Shotgun project has been deposited at GenBank under accession no. JBSTUI000000000. The version described in this paper is version JBSTUI010000000. The 16S rRNA gene sequence has been deposited in GenBank, and the accession no. is PX795507. The raw genome sequence data obtained on the Illumina NovaSeq 6000 platform and PacBio Sequel IIE platform have been deposited in SRA under accession nos. SRR35798209 and SRR37072457, respectively. The BioProject and BioSample accession nos. are PRJNA1335940 and SAMN52031148, respectively.
